# Protective effect of NSA on intestinal epithelial cells in a necroptosis model

**DOI:** 10.18632/oncotarget.21418

**Published:** 2017-09-30

**Authors:** Wei Dong, Min Zhang, Yaxi Zhu, Yuanhan Chen, Xingchen Zhao, Ruizhao Li, Li Zhang, Zhiming Ye, Xingling Liang

**Affiliations:** ^1^ Division of Nephrology, Guangdong General Hospital, Guangdong Academy of Medical Sciences, Guangzhou, China; ^2^ Department of Gastroenterology, The Sixth Affiliated Hospital of Sun Yat-Sen University, Guangzhou, China; ^3^ Department of Pathology, The Sixth Affiliated Hospital of Sun Yat-Sen University, Guangzhou, China

**Keywords:** intestinal epithelial cells, inflammatory bowel disease, colitis, necroptosis, necrosulfonamide

## Abstract

**Objective:**

This study aimed to investigate the protective effect of the necroptosis inhibitor necrosulfonamide (NSA) on intestinal epithelial cells using a novel *in vitro* necroptosis model that mimics inflammatory bowel disease (IBD).

**Methods:**

2,4,6-trinitrobenzenesulfonic acid (TNBS) was perfused into the rectum of BALB/c mice to established a colitis model. Pathologic injury and cell death were evaluated. A novel *in vitro* model of necroptosis was established in Caco-2 cells using TNF-*α* and Z-VAD-fmk, and the cells were treated with or without NSA. Morphologic changes, manner of cell death and the levels of phosphorylation of receptor-interacting protein kinase 3 (p-RIPK3) and mixed-lineage kinase domain-like (p-MLKL) were detected.

**Results:**

In the TNBS-induced colitis in mice, TUNEL-positive and caspase-3-negative cells were observed in the intestinal mucosa, and p-RIPK3 was found to be elevated. Under the stimulation of TNF-*α* and Z-VAD-fmk, the morphologic damage in the Caco-2 cells was aggravated, the proportion of necrosis was increased, and the level of p-RIPK3 and p-MLKL were increased, confirming that the regulated cell death was necroptosis. NSA reversed the morphological abnormalities and reduced necrotic cell death induced by TNF-*α* and Z-VAD-fmk.

**Conclusion:**

NSA can inhibit necroptosis in intestinal epithelial cells *in vitro* and might confer a potential protective effect against IBD.

## INTRODUCTION

Inflammatory bowel disease (IBD), which includes ulcerative colitis and Crohn’s disease, is a chronic inflammatory disorder of the gastrointestinal tract. Intestinal epithelial cells (IECs) play crucial roles in intestinal homeostasis. It has been speculated that dysregulation of cell death pathways in IECs is involved in IBD pathogenesis in humans [1].

It is accepted that tumor necrosis factor receptor-1 (TNFR1)-mediated apoptosis is the only type of regulated cell death [2]. When TNFR1 is activated, TNF receptor type 1-associated death domain (TRADD) protein and receptor-interacting protein kinase 1 (RIPK1) are recruited to TNFR1 to form a complex known as Complex I. Subsequently, RIPK1 dissociates from TNFR1 and recruits Fas-associated protein with death domain (FADD) and caspase 8 to form another complex known as Complex II. Then, activated caspase-8 activates downstream caspase-3, caspase-6, and caspase-7 to initiate apoptosis [3].

Recently, a TNFR1-mediated, caspase-independent mode of cell necrosis termed “necroptosis” was identified by the Nomenclature Committee on Cell Death [4]. Both necroptosis and apoptosis employ TNFR1 as a common upstream signal, but during apoptosis, caspase-8 cleaves receptor-interacting protein kinases (RIPKs) and inhibits necroptosis signals. If the caspase system is suppressed, RIPK1 recruits receptor-interacting protein kinase 3 (RIPK3) through the RIPK homotypic interaction motif (RHIM) domain promoting RIPK3 phosphorylation [5, 6]. Phosphorylated RIPK3 (p-RIPK3) mediates phosphorylation of mixed-lineage kinase domain-like (MLKL), ultimately leading to plasma membrane rupture [7, 8].

Mice with a conditional deletion of caspase-8 or FADD in the intestinal epithelium spontaneously undergo intestinal necroptosis and develop colitis [9, 10]. Moreover, recent reports have provided evidence of reduced caspase-8 with increased RIPK3 in inflamed tissues in IBD [11]. These studies identified the pathogenic effect of necroptosis during colitis. However, necroptosis has not been studied *in vitro* nor *in vivo* using non-genetic models of IBD. In the present study, we investigate necroptosis in TNBS-induced colitis and a novel *in vitro* model using IECs.

Necrosulfonamide (NSA) is a small-molecule compound shown to bind human MLKL and prevent plasma membrane rupture, which was identified through high-throughput screening of 200,000 compounds and subsequent studies on structure–activity relationships. Preliminary studies suggested that it can reduce necroptosis of certain cell types, including ovarian cells [12], dendritic cells [13] and acute myeloid leukemia cells [14]. However, no study has investigated the protective effect of NSA against intestinal epithelium necroptosis. In this study, we aim to investigate the protective effect of NSA against intestinal epithelium necroptosis using a novel *in vitro* model that mimics IBD.

## RESULTS

### RIPK3-dependent necroptosis in TNBS-induced colitis

**Figure 1:**
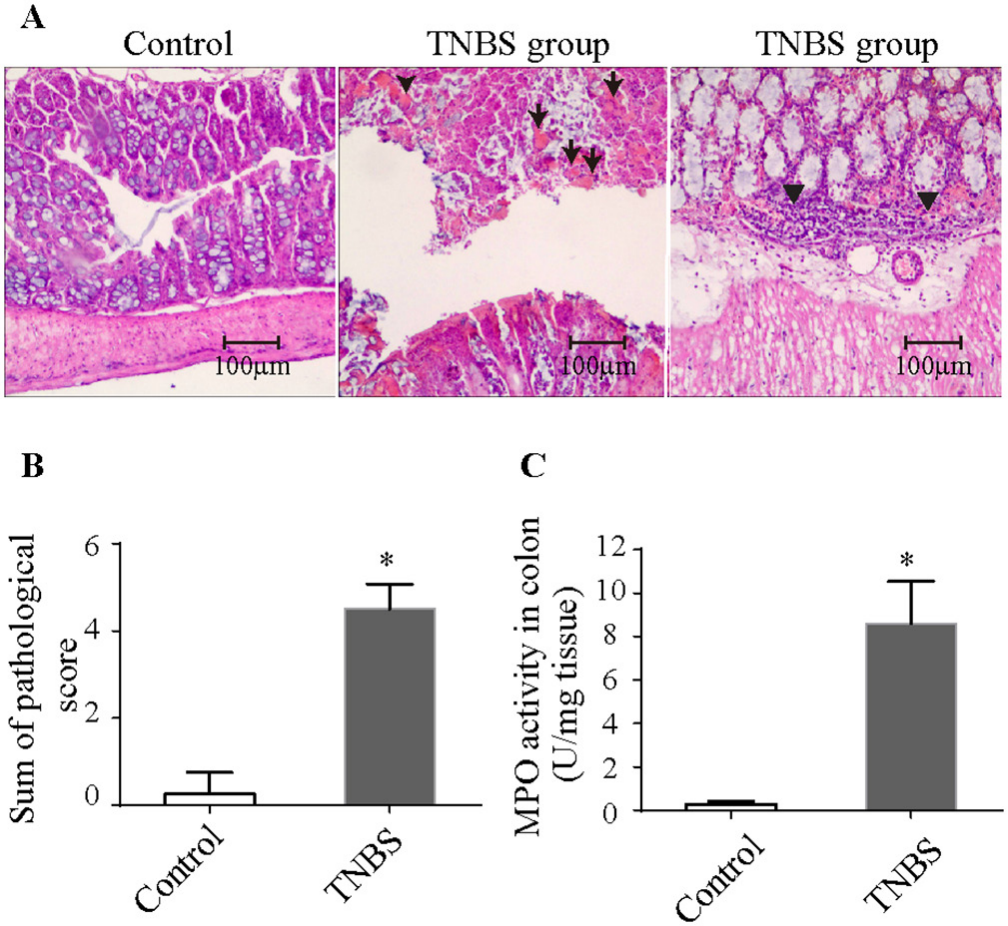
Histopathologic changes and MPO activity in TNBS-induced colitis **(A)** Histopathologic changes in the colonic tissues upon H&E staining (100× magnification). Arrows denote necrotic materials, and arrowheads denote intestinal infiltration by inflammatory cells. **(B)** Pathologic scores were elevated in TNBS-induced colitis. **(C)** MPO activity was increased in TNBS-induced colitis. The results are the mean ± standard deviation. *P<0.05 compared with the control group. TNBS, 2,4,6-trinitrobenzenesulfonic acid; MPO, Myeloperoxidase.

TNBS-induced colitis was characterized by mucosal erosion, desquamated glandular epithelial cells, intestinal interstitial congestion, focal infiltration of inflammatory cells and necrotic materials (Figure 1A). The pathologic injury score and MPO activity in the colitis group were significantly higher than those of the control group (Figure 1B & 1C).

**Figure 2:**
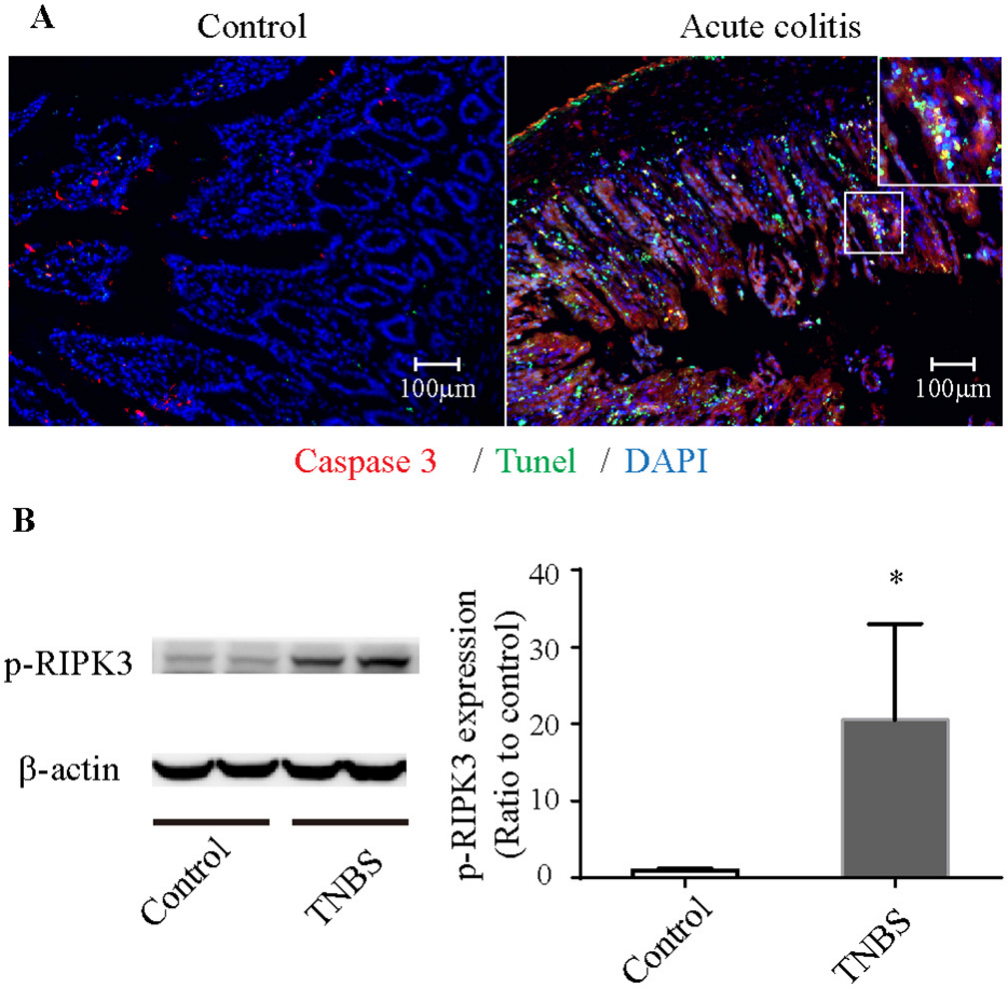
Necroptosis in TNBS-induced colitis **(A)** Immunofluorescence of colonic tissues stained with cleaved caspase-3 (red), TUNEL (green) and DAPI (blue). The enlarged image shows green fluorescence representing TUNEL-positive and caspase-3-negative necroptotic cells and yellow fluorescence representing TUNEL-positive and caspase-3-positive apoptotic cells. **(B)** Western blot analysis of p-RIPK3. p-RIPK3 is increased in TNBS-induced colitis. *P<0.05 compared with the control group. TNBS, 2,4,6-trinitrobenzenesulfonic acid; p-RIPK3, Phosphorylated receptor-interacting protein kinase 3.

Immunofluorescence revealed a significant number of TUNEL-positive and caspase-3-negative cells in the intestinal mucosa in the colitis group (Figure 2A). WB analysis showed that the level of p-RIPK3 in colitis group was also increased (Figure 2B). These results indicated that necroptosis was involved in the TNBS-induced colitis.

**Figure 3:**
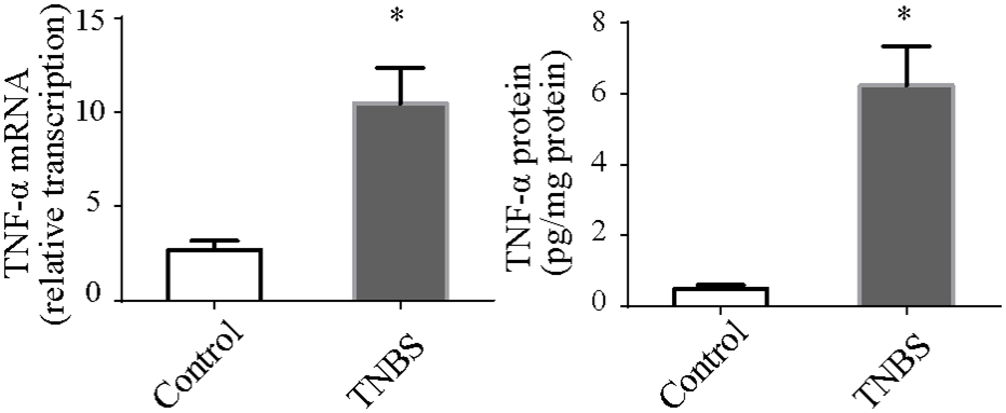
TNF-α mRNA and protein in colonic tissues TNF-α mRNA and protein levels were both up-regulated in TNBS-induced colitis. *P<0.05 compared with the control group.

To understand the signaling pathways in IEC necroptosis, we measured TNF-α levels in the intestine. In the colitis group, the intestinal levels of TNF-α were significantly increased (Figure 3). This suggested that TNF-α plays a pathogenetic role in the necroptosis of intestinal tissue.

### Morphologic damage to Caco-2 cells upon treatment with TNF-α plus Z-VAD-fmk

**Figure 4:**
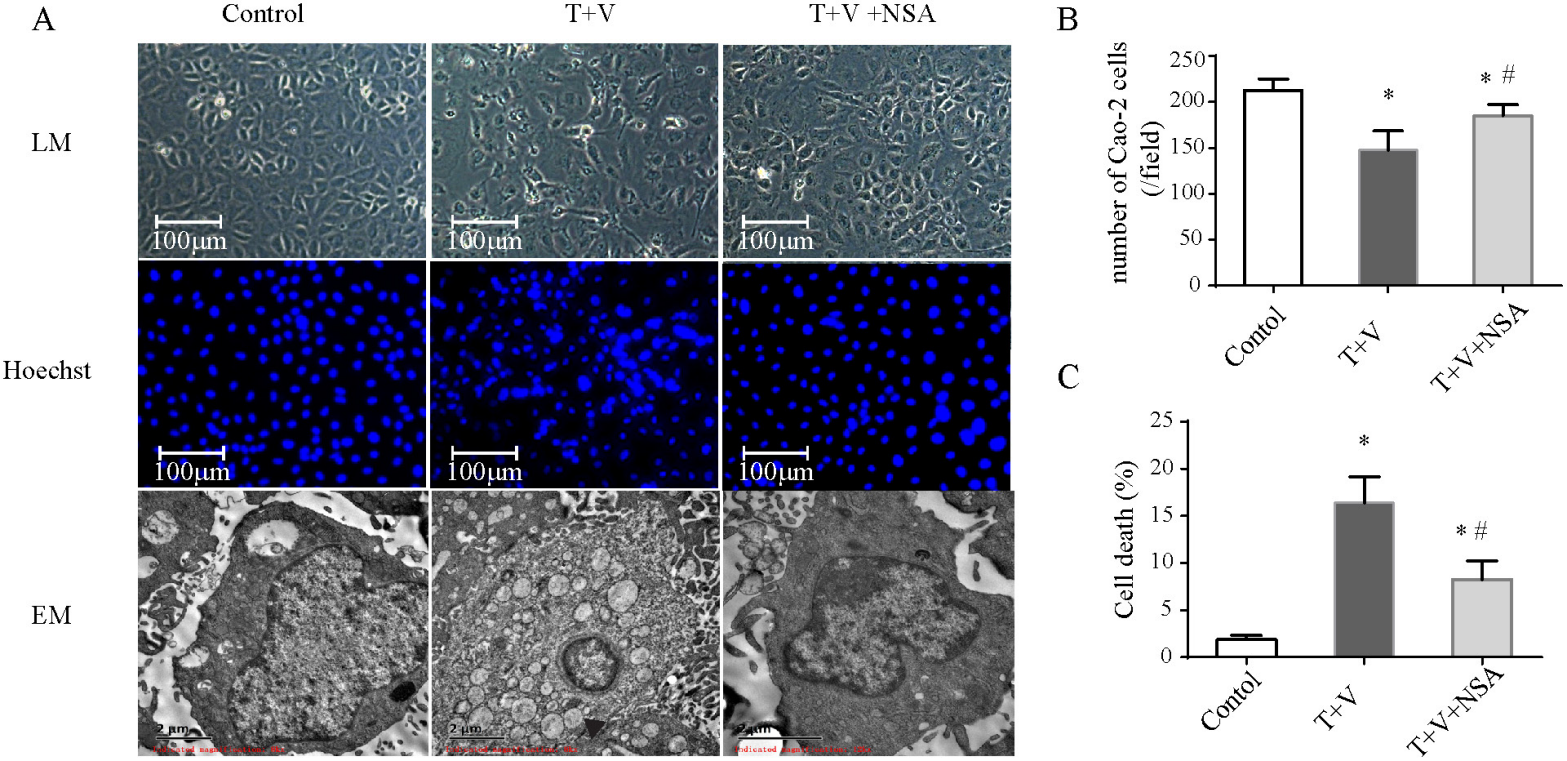
Morphologic changes in Caco-2 cells treated with TNF-α (T) and Z-VAD-fmk (V) with or without necrosulfonamide (NSA) **(A)** Cells under different conditions were observed with a light microscope (LM) for morphologic change, a fluorescence microscope for Hoechst-dye fluorescence, and an electron microscope (EM) for ultrastructural change. Cells became elongated and/or enlarged, and some cells were detached. Hoechst dye showed increasing irregular cell nuclei, nuclear condensation and fragmentation. Cellular swelling, organelle swelling and plasma membrane rupture were observed under the electron microscope. **(B)** Quantification of Cao-2 cells number in each group. **(C)** Quantification of cell death which was indicated by Hoechst 33258 Stains. *P<0.05 compared with the control group; # P<0.05 compared with the T+V group.

Because TNF-α activation and caspase inhibition are both essential for IEC necroptosis, we established a necroptosis model in Caco-2 cells using TNF-α plus Z-VAD-fmk (T+V group), which was similar to the necroptosis model in renal tubular epithelial cells in our previous study [15]. Caco-2 cell numbers were reduced, and the cells exhibited irregular shapes in the T+V group (Figure 4A, 4B). The cells become elongated and/or enlarged, and some cells became detached from the underlying matrix. Fluorescence using Hoechst dye showed irregular cell nuclei, nuclear condensation and fragmentation are increased (Figure 4A). These morphology change meant increasing cell death (Figure 4C). Electron microscopy showed characteristics of necrosis including cellular swelling, swelling of organelles and plasma membrane rupture (Figure 4A).

### RIPK3-dependent cell necrosis upon treatment with TNF-α plus Z-VAD-fmk

**Figure 5:**
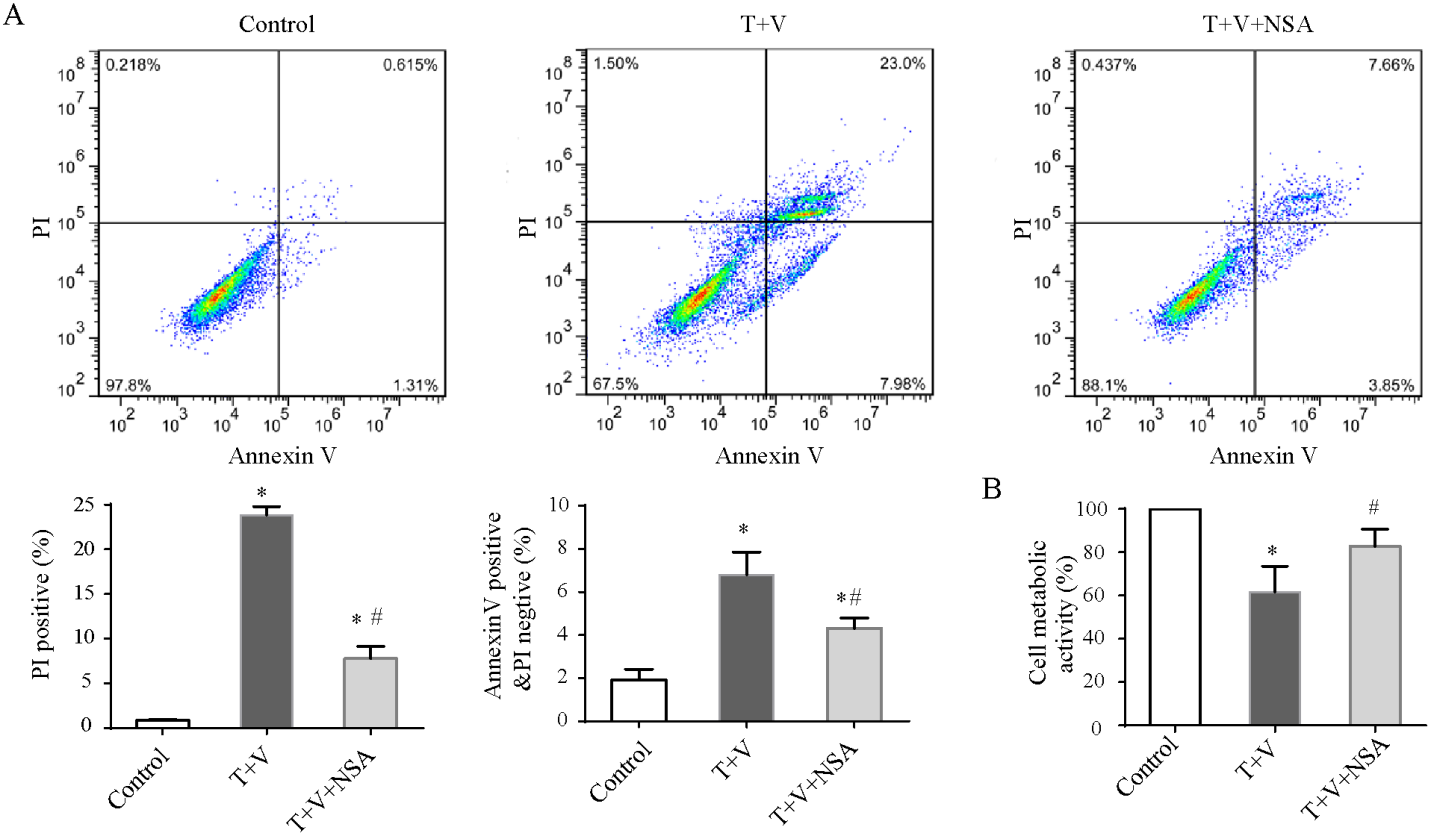
Regulated necrosis and early apoptosis of Caco-2 cells Caco-2 cells were treated with TNF-α (T) and Z-VAD-fmk (V) with or without necrosulfonamide (NSA). **(A)** Typical flow cytometric graph of necrotic cells under different conditions, and histogram showing proportions of propidium iodide (PI)-positive cells or Annexin V-positive PI negative cells. Data in the histogram represent four experiments. **(B)** Cell metabolic activity was measured by the 3-(4,5-Dimethylthiazol-2-yl)-2,5-diphenyltetrazolium bromide (MTT) assay and expressed as the percentage of control. Data in the histogram represent four experiments. The results are the mean ± standard deviation. *P<0.05 compared with the control group; # P<0.05 compared with the T+V group.

**Figure 6:**
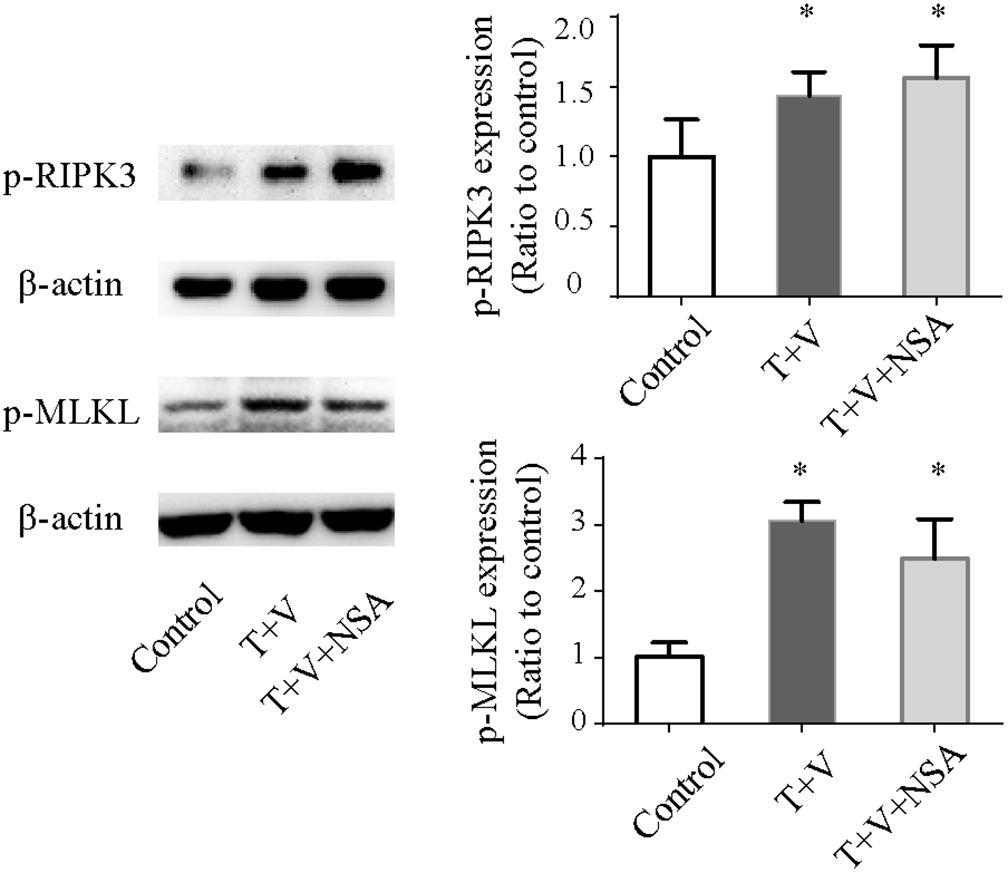
Western blot analysis of p-RIPK3 and p-MLKL in Caco-2 cells treated with TNF-α (T) and Z-VAD-fmk (V) with or without necrosulfonamide (NSA) *P<0.05 compared with the control group. p-RIPK3, phosphorylated receptor-interacting protein kinase 3; p-MLKL, phosphorylated mixed-lineage kinase domain-like.

Flow cytometric analyses were used to determine necrotic death in Caco-2 cells by measuring PI uptake. Treatment with TNF-α plus Z-VAD-fmksignificantly induced cell necrosis (Figure 5A). We used the MTT assay to detect cellular metabolic activity to reflect cellular death as has been previously reported. Treatment with TNF-α plus Z-VAD-fmk could attenuate cellular metabolic activity (Figure 5B). Moreover, Western blot analysis showed that the level of p-RIPK-3 and p-MLKLwas increased in the T+V group (Figure 6). These results indicated that treatment with TNF-α plus Z-VAD-fmk may be able to induce necroptosis in Caco-2 cells.

### Protective effect of NSA on Caco-2 cells upon treatment with TNF-α plus Z-VAD-fmk

NSA markedly decreased the morphologic damage and the number of detached cells (Figure 4) and improved metabolic activity compared with those in the T+V group (Figure 5B). Although NSA administration did not reduce the level of p-RIPK-3 and p-MLKL, NSA decreased the number of PI-positive Caco-2 cells due to the inhibition of MLKL function. In addition, NSA also reduced early apoptosis which were Annexin-V positivity and PI negativity (Figure 5A). Using electron microscopy, we found that NSA also reversed the morphological abnormalities induced by TNF-α plus Z-VAD-fmk. Considering the results mentioned above, NSA could significantly protect Caco-2 cells from necroptosis.

## DISCUSSION

In the model of TNBS-induced colitis, we observed cell death characterized by necrosis (TUNEL-positive and caspase-3-negative) and elevated p-RIPK3 in the intestinal mucosa. The results of our animal experiment were similar with those of mouse models with a conditional deletion of caspase-8 or FADD in the intestinal epithelium [9, 10]. This indicated that necroptosis is involved in TNBS-induced colitis. We also found that the intestinal levels of TNF-α were significantly increased, suggesting that TNF-α plays a pathogenetic role in the necroptosis of intestinal tissue.

TNF-α and Z-VAD-fmk are irritants used to generate an *in vitro* model of necroptosis. TNF-α is used to stimulate TNFR1 activation. Caspase-8 prevents RIP3-dependent necroptosis through the disruption of formation of a stable necrosome complex [16], and the levels of caspase-8 are reduced in the inflamed tissues of patients with active IBD [11]. Therefore, TNF-α and Z-VAD-fmk can mimic the conditions observed in IBD. We established a novel *in vitro* model of necroptosis in IECs by treatment with TNF-α plus Z-VAD-fmk. In the T+V group, an increase in the number of necrosis cells and elevated p-RIPK3 were observed, which are both characteristics of necroptosis.

When TNFR1 is activated and the caspase system is suppressed, RIPK-1 phosphorylates RIPK3 forming a ‘necrosome’, and the ‘necrosome’ subsequently phosphorylates MLKL, resulting in necroptosis [3]. In a model of spontaneous colitis using FADD-knockout mice, RIPK3 knockout prevented the development of spontaneous disease in the small intestine and colon [9]. Compared with gene modification techniques, the use of a necroptosis inhibitor is easier for translation ‘from bench to bedside’. Although one study had reported that necrostatin-1, a RIPK1 kinase inhibitor, reduces intestinal inflammation [17], studies on the protective effect of necroptosis inhibitors against colitis are few. NSA is a small molecule that binds to MLKL and prevents plasma membrane rupture, identified through high-throughput screening of 200,000 compounds and subsequent studies on structure–activity relationships [8]. Although NSA can protect several human cell types from necrosis [12–14], no study has investigated the protective effect of NSA against intestinal epithelium necroptosis. Because NSA targets the Cys86 residue of human MLKL and not mouse MLKL [8], we did not test protective effect of NSA against TNBS-induced colitis in mice; hence, we explored the protective effect of NSA on Caco-2 cells in a novel *in vitro* model of necroptosis. In our study, NSA could improve cellular morphology and metabolic activity and eventually reduce necroptosis induced by TNF-α and Z-VAD-fmk. We have examined the phosphorylation of RIPK3 and MLKL, and found that the level of p-RIPK3 and p-MLKL were increased in the T+V group. However, NSA did not reduce the level of p-RIPK3 and p-MLKL, which might due to that NSA does not affect phosphorylation of MLKL but targets the Cys86 residue of MLKL blocking translocation of phosphorylated MLKL to cell membrane [7].

Theoretically, NSA does not affect apoptosis because NSA inhibits MLKL function which is not involved in apoptosis. However we found NSA also reduced early apoptosis. There might be two explanations for protective effect of NSA on apoptosis. First, necrosis cells release DAMPs such as HMGB-1 [18], which could induce apoptosis [19, 20]. When necrosis was inhibited, release of DAMPs was reduced and apoptosis was attenuated indirectly. Secondly, NSA might alleviate apoptosis by its off-target effects.

The end result of necroptosis is necrosis, and necroptosis is a strong trigger of inflammatory responses, resulting in more severe damage to tissues than that observed due to apoptosis [21]. If necroptosis-inhibiting drugs similar to NSA can be used in clinical practice, they may help alleviate the symptoms of IBD. Considering the side effects of TNF-α inhibitors and some TNF-α resistant cases, blocking the function of MLKL may be valuable for the development of adjuvant therapies for IBD.

In conclusion, the MLKL blocker NSA can inhibit necroptosis in intestinal epithelial cells *in vitro*, and it may be used as a new potential therapeutic strategy in IBD.

## MATERIALS AND METHODS

### 2,4,6-trinitrobenzenesulfonic acid (TNBS)-induced model of colitis

All protocols involving animals were in accordance with the regulations set by the Ethics Committee of the Sixth Affiliated Hospital of Sun Yat-sen University (Guangzhou, China).

BALB/c mice were obtained from the Laboratory Animal Center of Sun Yat-sen University and maintained under standard conditions. Sixteen male mice (8- to 10-week-old) were divided randomly into 2 groups of eight: control group and colitis group. TNBS-induced colitis was established as described previously with minor modifications [22]. In brief, after fasting the mice for 24 h, a vinyl catheter was inserted (to a depth of 3 cm) into the anus of mice, and 2 mg TNBS (Sigma–Aldrich, Saint Louis, MO, USA) in 50% ethanol was perfused into the rectum, followed by another perfusion after 7 days. The mice were euthanized 3 days after the second perfusion to examine injury to the colon.

### Pathology

Formalin-fixed colon segments were embedded in paraffin, and sections (5 μm thick) were stained with hematoxylin and eosin (H&E). The pathologic injury of colitis was scored by an observer blinded to the study protocol.

Injury to tissue due to colitis was evaluated using a scoring system that combines macroscopic and microscopic histopathologic scores. The macroscopic scoring system was as follows: normal, 0; focal ulceration, 1; multifocal ulceration, 2; diffuse ulceration and necrosis, 3. The microscopic histopathologic scoring system was as follows: normal, 0; inflammation in the mucosa and submucosa; 2, inflammation in the entire wall of the intestine and 3, ulceration and necrosis in the entire wall [23]. The sum of the macroscopic and microscopic histologic scores was regarded as the total score for each mouse.

### Myeloperoxidase (MPO) activity

MPO activity was used as a marker of neutrophil infiltration. MPO activity in tissue was determined using an MPO detection kit (Jiancheng Bioengineering Institute, Nanjing, China) as previously described. This method is based on the MPO-catalyzed hydrogen peroxide-mediated oxidation of *o*-dianisidine [24]. The OD (optical density) of the yellow oxidation product was measured at 460 nm. One unit of MPO activity was defined as the amount of enzyme that degraded 1 μM of hydrogen peroxide per min at 25°C.

### Immunofluorescence staining

Necrosis is independent of caspase, so terminal deoxynucleotidyl transferase (TdT) dUTP nick-end labeling (TUNEL)-positive and caspase-3-negative cells were used as markers of necrosis in the intestinal tissue [10, 25]. For double labeling with cleaved caspase-3, TUNEL staining was performed in a series of sections. After that, DAPI was used to stain the nuclei. Immunofluorescence reactivity was viewed on a fluorescence microscope (Zeiss Axio Imager Z1, Germany).

### Measurement of TNF-α levels in the colonic tissue

Samples of fresh tissue (250–500 mg) were homogenized in 10× vol of cold potassium buffer, and levels of TNF-α protein were determined using enzyme-linked immunosorbent assay (Abcam, Shanghai, China). Total RNA from intestinal tissues was extracted with TRIzol^®^ Reagent (Invitrogen, Carlsbad, CA, USA) according to the manufacturer’s instructions. The level of TNF-α mRNA was determined by quantitative real-time reverse transcription-polymerase chain reaction (qRT-PCR) using Power SYBR Green PCR Master Mix (TaKaRa Biotechnology, Shiga, Japan) and a 7500 qRT-PCR system (Applied Biosystems, Foster City, CA, USA). The housekeeping gene β-actin was used as the internal control. The primers specific for TNF-α and β-actin used for real-time qRT-PCR were as follows (forward and reverse, respectively): 5′-AAGAGGCACTCCCCCAAAAGAT-3′ and 5′-TCTGAGTGTGAGGGTCTGGGC-3′; 5′-TGGAATCCTGTGGCATCCATGAAAC-3′ and 5′-TAAAACGCAGCTCAGTAACAGTCCG-3′. Each reaction was performed in triplicate, and the fold change in the expression of each gene was calculated using the 2^−ΔΔCT^ method.

### IEC culture

Human epithelial colorectal adenocarcinoma (Caco-2) cells (American Type Culture Collection, Manassas, VA, USA) were seeded in 75 cm^2^ culture plates in a humidified atmosphere of 5% CO_2_ at 37°С. Cells were maintained in high-glucose (4.5 g/L) Dulbecco’s modified Eagle’s medium (DMEM; Sigma–Aldrich) supplemented with 10% heat-inactivated fetal bovine serum (FBS; Gibco, Waltham, MA, USA), 50 U/mL penicillin and 50 μg/mL streptomycin. To induce necroptosis, cells were treated with TNF-α and Z-VAD-fmk. The latter can completely block the caspase cascade and promote the conversion from apoptosis to necrosis in IECs. NSA was used to block the function of MLKL. The cells were divided into three groups: (i) control (cells were maintained in DMEM supplemented with 10% FBS; (ii) T+V (cells were treated with 10 ng/mL TNF-α (PeproTech, Suzhou, China) and 50 μM Z-VAD-fmk (Sigma-Aldrich); (iii) T+V+NSA (cells were treated with 10 ng/mL TNF-α, 50 μM Z-VAD-fmk and 2 μM NSA (Toronto Research Chemicals, Toronto, Canada). All experiments were performed in an atmosphere of 5% CO_2_ for 24 h at 37°C.

### Hoechst 33258 Stains

Cao-2 cells were seeded on sterile cover glasses placed in the six-well plates at a density of 1.0 × 10^4^ cells cm^−2^ and cultured at 37°C under different experimental treatments. Subsequently, cells were fixed, washed twice with PBS, and stained with Hoechst 33258 staining solution according to the manufacturer’s instructions (Beyotime, Shanghai, China). Stained nuclei were observed under fluorescence microscope (Zeiss Axio Imager Z1). Typical dead cells were identified by morphology including nuclear condensation and fragmentation. Four fields with ∼200 cells per field were examined in each condition to estimate the percentages of cell death.

### Mitochondrion-dependent metabolic activity

IECs under different experimental conditions were harvested for 3-(4,5-Dimethylthiazol-2-yl)-2,5-diphenyltetrazolium bromide (MTT) assays (Nanjing KeyGen Biotech, Nanjing, China) to determine the mitochondrion-dependent metabolic activity. Briefly, 3×10^5^ cells were plated in a 96-well plate (100 μL for each well) for 24 h, followed by the different treatments mentioned above. Then, 100 μL of MTT dye solution was added to the wells containing 100 μL of growth medium. After gentle shaking overnight at 37°C, MTT reduction was quantified spectrophotometrically at 570 nm using a microplate reader (680; Bio-Rad Laboratories, Berkeley, CA, USA). The assay was repeated six times. Metabolic activity (%) was calculated as follows:

Metabolic activity (%)=[OD (experiment)−OD (blank)]/[OD (control)−OD (blank)]×100%.

### Flow cytometry

Necrotic cells were identified using an Annexin V/propidium iodide (PI) detection kit according to the manufacturer’s instructions (Nanjing KeyGen Biotech, Nanjing, China). Briefly, the cell pellet (including floating cells) was resuspended in 1× binding buffer, followed by incubation with 5 μL of Annexin V-FITC and 5 μL of PI in the dark for 10 min. Cell fluorescence was analyzed using a flow cytometer (FACSAria II; BD Biosciences, San Diego, CA, USA).

### Transmission electron microscopy

The cells were trypsinized and washed with phosphate-buffered saline, and then cell pellets were fixed with 2% glutaraldehyde in 0.15 mM cacodylate buffer (pH 7.2) at 4°C for 1 h and washed in 0.15 mM cacodylate buffer (3×; 5 min). Following that, the cell pellets were fixed in 1% osmium tetroxide at 4°C for 30 min, rinsed with dH_2_O at 4°C (3×; 2 min) and stained with 1% aqueous uranyl acetate at 4°C for 30 min. Finally, the cell pellets were dehydrated in an ethanol gradient and embedded in resin (Epon 812) for 12 h. Following 12 h in the oven at 60°C for polymerization, the samples were cut into extremely thin sections (1 μm), collected and placed on a small circular metal grid prior to being viewed. Transmission electron microscopy was performed using a JEM-100CX system (JEOL, Tokyo, Japan) to observe cellular morphology and capture images of cellular ultrastructures.

### Western blotting

Cells under different experimental conditions or freshly isolated intestinal tissues were harvested and lysed immediately with cytoplasm extraction buffer. Protein concentration was quantified using a Bradford assay kit (Thermo Fisher Scientific, Carlsbad, CA, USA). Total cell lysates or tissue lysates were separated by sodium dodecyl sulfate–polyacrylamide gel electrophoresis and transferred to polyvinylidene difluoride membranes (Amersham Biosciences, Buckinghamshire, UK) for blotting with appropriate primary antibodies against β-actin (#8457 at 1:1000, Cell Signaling Technology, Inc., Danvers, MA, USA), human RIPK-3 phospho-S227 (ab209384 at 1:2000, Abcam), human MLKL phospho-S358 (ab187091 at 1:1000, Abcam) and mouse RIPK3 phospho-S232 (ab195117 at 1:1000, Abcam). After being washed in TBS, the blots were incubated with horseradish peroxidase-conjugated goat anti-rabbit antibodies (#7077 at 1:2000, Cell Signaling Technology) for 1 h at room temperature. After a final wash as described above, the corresponding secondary antibodies were visualized using enhanced Pierce™ ECL Western Blotting Substrate (Thermo Fisher Scientific). Signals were quantified with a chemiluminescence detector and the accompanying densitometry software (Tanon, Shanghai, China).

### Statistical analyses

All values are expressed as mean ± standar, d deviation (SD). Multiple comparisons among the groups were conducted by one way Analysis of variance (ANOVA) followed by least square difference (LSD) multiple comparison test. All statistical analyses were performed using SPSS software (version 21.0; IBM SPSS, Armonk, NY, USA), with values of P < 0.05 considered to be statistically significant.

## Abbreviations

NSA: Necrosulfonamide
RIPK: receptor-interacting protein kinase
IBD: Inflammatory bowel disease
IECs: Intestinal epithelial cells
TNFR1: tumor necrosis factor receptor-1
MLKL: mixed-lineage kinase domain-like
TNBS: 2,4,6-trinitrobenzenesulfonic acid
T+V: TNF-α plus Z-VAD-fmk
